# Unsupervised Anomaly Detection Framework for Multimodal Data in Industrial Control Systems

**DOI:** 10.3390/s26123914

**Published:** 2026-06-20

**Authors:** Yunsung Kim, Gyeongdeok An, Kihyun Kim, Jaecheol Ha

**Affiliations:** 1 Department of Information Security, Hoseo University, Asan-Si 31499, Republic of Korea; veluv@naver.com (Y.K.); akg13078@gmail.com (G.A.); 2 NNSP Co., Ltd., 100, Nonhyeon-Ro, Gangnam-Gu, Seoul-Si 06303, Republic of Korea; khkim@nnsp.co.kr

**Keywords:** industrial control system (ICS), anomaly detection, multimodal data, sensor–network fusion, unsupervised learning, autoencoder, time synchronization, cross-modal complementarity

## Abstract

Industrial control systems (ICSs) are cyber–physical environments in which physical process data and network communication data are generated simultaneously. Existing studies have mainly focused on either sensor-based or network-based anomaly detection, making it difficult to capture diverse attack indicators and motivating the use of multimodal methods that can leverage complementary information from both modalities. In this paper, we propose an unsupervised multimodal anomaly detection framework for ICSs that jointly uses sensor and network modalities. For each modality, autoencoder-based single-modality models are trained in an unsupervised manner, and their anomaly scores and latent feature vectors are extracted. These outputs are temporally aligned to construct a time-aligned multimodal table, which is then used to implement and compare two fusion strategies: anomaly score fusion and latent feature fusion. In latent feature fusion, aligned modality-specific latent features are combined with canonical correlation analysis (CCA)-derived cross-modal correlation features. The experimental results showed that latent feature fusion achieved stable performance across multiple sensor–network encoder combinations. In particular, the gated recurrent unit–convolutional neural network (GRU–CNN) combination achieved the best F1-score of 0.9166 and ROC-AUC of 0.9795. In addition, the complementarity analysis showed that latent feature fusion recovered some missed detections by integrating complementary sensor and network evidence. These results demonstrate that latent feature fusion is an effective multimodal strategy for ICS anomaly detection.

## 1. Introduction

Industrial control systems (ICSs) monitor and control physical processes across critical national industries, including power, water treatment, and manufacturing. The system incorporates sensors and actuators, programmable logic controllers (PLCs), distributed control systems (DCSs), supervisory control and data acquisition (SCADA) systems, human–machine interfaces (HMIs), and the industrial networks that connect them [1]. In contrast to conventional IT systems, ICSs place greater emphasis on availability, operational continuity, and safety than on confidentiality [2]. This results in fundamental differences in the design philosophy and operating environment. Furthermore, the proliferation of Industry 4.0 and the Industrial Internet of Things (IIoT) has expanded the connectivity and attack surface of ICSs, increasing the need for efficient and lightweight intrusion detection in highly connected environments [3]. In this context, security incidents in ICSs can result in a multitude of adverse consequences, including information leakage, equipment damage, process disruption, and even human and societal harm [4].

ICS anomaly detection can be categorized broadly into physics- and network-based detection depending on the type of data used [5]. Physics-based anomaly detection identifies anomalies using process-time-series sensor data such as water level, flow rate, pressure, and temperature, directly reflecting the abnormalities in the physical process. Nevertheless, anomalies caused by sensor faults or environmental changes are difficult to distinguish from those caused by cyberattacks [6]. Network-based anomaly detection identifies attack-related anomalies by analyzing traffic and its correlation within industrial protocols, such as Modbus, DNP3, S7Comm, and PROFINET, enabling the faster detection of malicious activities. On the other hand, it is difficult to directly explain how abnormal communication has affected the actual physical process [7].

Against this background, recent studies have begun to explore anomaly detection approaches that jointly leverage multiple data sources or modalities in ICS environments [8]. Here, multimodal data refer to data in which sensor-based physical process data and network communication data record the same ICS operational state from different perspectives. Multimodal detection is expected to overcome the limitations of single-modality detection by simultaneously considering the changes in physical process states and behaviors at the network communication layer.

This paper proposes a sensor network-based multimodal unsupervised anomaly detection framework for ICSs. In the proposed framework, each sensor and network modality is first trained independently and unsupervised using autoencoder-based models. The anomaly scores and latent features are extracted from each modality. The two modalities are then synchronized by constructing common time points through timestamp alignment. In light of the synchronized data, multimodal anomaly detection is performed using anomaly score fusion and latent feature fusion. In the latent feature fusion path, modality-specific latent features are combined with correlation-related information between the sensor and network modalities. This framework shows that multimodal detection can compensate for the limitations inherent to single-modality detection. Furthermore, a systematic comparison and analysis of the detection characteristics of different fusion strategies and the complementarity of multimodal detection is presented.

The major contributions of this paper are as follows:

- This paper proposes a multimodal unsupervised anomaly detection framework for ICSs that combines sensor and network modalities.
- Anomaly score fusion and latent feature fusion were implemented based on the anomaly scores and latent features extracted from single-modality autoencoders for sensors and networks, and the two fusion strategies were compared under the same experimental conditions.
- The proposed framework was evaluated in terms of detection performance, robustness across repeated random seeds, and cross-modal complementarity, showing that sensor and network modalities provide complementary anomaly evidence in ICS environments.

The remainder of this paper is organized as follows. Section 2 reviews previous studies on ICS anomaly detection. Section 3 describes the dataset, data quality assessment, and data split. Section 4 presents the proposed multimodal unsupervised anomaly detection framework. Section 5 describes the experimental setup and evaluation methodology, followed by an analysis of the detection results. Finally, Section 6 concludes the paper and discusses future research directions.

## 2. Related Work on ICS Anomaly Detection

### 2.1. Anomaly Detection in ICS

Research on ICS anomaly detection can be broadly divided into sensor-based anomaly detection for physical processes and network-based anomaly detection for communication behavior, depending on the characteristics of the data used. The former detects anomalies from the changes in sensors, actuators, and process variables, whereas the latter identifies abnormal behavior from communication data such as packets, sessions, and protocol fields.

#### 2.1.1. Sensor-Based Anomaly Detection

Sensor-based anomaly detection is considered the most common approach because it directly analyzes multivariate time-series data measured from the physical processes of ICSs. In particular, unsupervised learning has been widely adopted because attack labels are often difficult to obtain in real industrial environments [9]. In this setting, models are trained only on normal data to learn normal patterns, and deviations from normal behavior are identified as anomalies.

Early sensor-based approaches often relied on statistical thresholds or prediction errors. In addition, machine learning and deep learning methods capable of modeling nonlinear dependencies in multivariate time series have become increasingly important as ICS data have become more complex [10]. In particular, among time-series models, the long short-term memory (LSTM) family is used widely as a core foundation for sensor anomaly detection because of its strength in learning long-term dependencies. Malhotra et al. [11] proposed a method for detecting anomalies by modeling the prediction errors of a stacked LSTM predictor trained on normal time series with a multivariate Gaussian distribution. This study became one of the representative early LSTM-based anomaly detection works. More recently, in addition to recurrent architectures, convolution-based time-series models have attracted attention. Gopali et al. [12] compared and analyzed temporal convolutional networks (TCNs) and LSTMs in a water treatment testbed and reported that ICS sensor data contain simple sequential information and local patterns, and multi-scale temporal structures.

In summary, sensor-based unsupervised anomaly detection has evolved through approaches such as reconstruction-error-based time-series models that reflect the spatiotemporal structure and deep models that capture local patterns. On the other hand, these methods inherently detect anomalies only after the changes in the physical process have been observed. This imposes a limitation; when an anomaly first occurs at the network layer, detection can be difficult in the early stages before its effects are sufficiently reflected in the process variables. In addition, these methods are sensitive to sensor noise and data quality, which imposes a structural limitation in distinguishing simple malfunctions from anomalous signals caused by cyberattacks.

#### 2.1.2. Network-Based Anomaly Detection

Network-based anomaly detection identifies abnormal behavior using communication data such as industrial protocol packets, sessions, and network flows. Unlike sensor-based detection, this approach focuses on identifying abnormal patterns at an early stage, emphasizing its potential as an early warning mechanism for anomaly detection [13].

Early studies mainly modeled the sequences of normal traffic or the input–output relationships between control and process behaviors. Song and Liu [14] extracted behavior-based sequences from Modbus/TCP traffic, constructed normal behavior-based models of the controller and the controlled process, and proposed a method for detecting anomalies by comparing real-time observed behavior with predicted behavior. Recently, autoencoder-based approaches that reconstruct normal network patterns have been actively studied. Zare et al. [15] proposed a sequence-to-sequence LSTM autoencoder model for detecting data manipulation attacks in network log data from a Modbus/TCP-based SCADA environment. This method showed that simple categorical packet features and the temporal structure of industrial protocol payloads are important.

In summary, network-based unsupervised anomaly detection has evolved toward learning normal communication patterns and detecting anomalies at the packet, protocol field, session, or flow level, including abnormal sequences and protocol field manipulation. This approach can enable earlier detection than sensor-based methods, potentially before changes appear in the physical process. On the other hand, because it does not directly reflect the semantics of the physical process, network-based anomaly detection alone has limitations in determining whether packet-level abnormalities actually lead to anomalies in the underlying process.

### 2.2. Multimodal Anomaly Detection and Fusion Strategies

Along with these existing studies, recent research on ICS anomaly detection has begun to explore multimodal approaches that jointly utilize sensor process and network modalities. Multimodal approaches are particularly effective in ICS environments because they learn by integrating information from different modalities. Such approaches can compensate for the limitations of single-modality detection and effectively leverage the complementary information from the two modalities by simultaneously reflecting changes in physical process states and abnormal behavior at the communication layer [16]. In multimodal data fusion research, fusion strategies are generally categorized according to the stage at which fusion is performed: early fusion, which directly integrates modalities at the input stage; late fusion, which integrates the individual detection results or prediction scores of each modality at the final stage; and intermediate fusion, which combines the latent features extracted by modality-specific encoders at an intermediate stage [17].

First, early fusion synchronizes the sensor and network modalities from the input stage, which is then used as the input to a single model. Du et al. [18] pointed out that a single dataset is insufficient to detect anomalies. Therefore, they constructed a cyber–physical dataset by combining the features from the two modalities and performed unsupervised anomaly detection using an LSTM autoencoder and a GAN-based model. Late fusion first operated independent anomaly detectors for each modality and then integrated the results at the final decision stage. Canonico et al. [19] explained that, in CPS security, analyzing only a single data source may fail to detect attacks that leave only weak traces in a specific stream. They first evaluated anomaly detection models that process the network and sensor modalities separately and then proposed a framework that aligns the temporal scales of the two modalities and generates the final alarm through decision fusion. This approach preserves the unique characteristics of each modality and remains applicable even when the structures differ substantially.

Intermediate fusion first extracts the latent features using modality-specific encoders and then combines them for further learning and anomaly detection. Zhan et al. [20] proposed an anomaly detection method that jointly models sensor and network data on a cross-domain graph and learns joint features and domain-specific features. Their method employs attention-based graph convolutional networks to learn both domain-shared and domain-specific representations, thereby capturing cross-domain relationships between physical process states and network behaviors. More recently, Pinto et al. [21] proposed an unsupervised multimodal anomaly detection framework that extracts latent representations from sensor and network data using VAE-LSTM encoders and fuses them through a dual cross-attention mechanism. Their approach also uses a discriminator-based adversarial component to regularize reconstruction and improve subtle anomaly detection. These studies indicate that intermediate fusion can be extended toward attention-based cross-domain representation learning.

In summary, multimodal anomaly detection has increasingly been studied as a way to address the limitations of sensor- and network-based single-modality approaches. Recent studies have examined how features from different modalities can be represented, aligned, and fused at different stages of the detection pipeline. These efforts indicate that multimodal anomaly detection in ICSs is still being explored from multiple methodological perspectives rather than converging on a single fixed fusion strategy.

## 3. Dataset for Deep Learning

### 3.1. SWaT Testbed

The Secure Water Treatment (SWaT) dataset [22] was collected from a scaled-down water treatment plant testbed developed at the iTrust Lab of the Singapore University of Technology and Design (SUTD). SWaT is a water treatment system that implements a reduced-scale version of a real water treatment plant and simulates the entire purification process through six sequential stages, from raw water intake to reverse osmosis and membrane backwashing.

**Figure 1:**
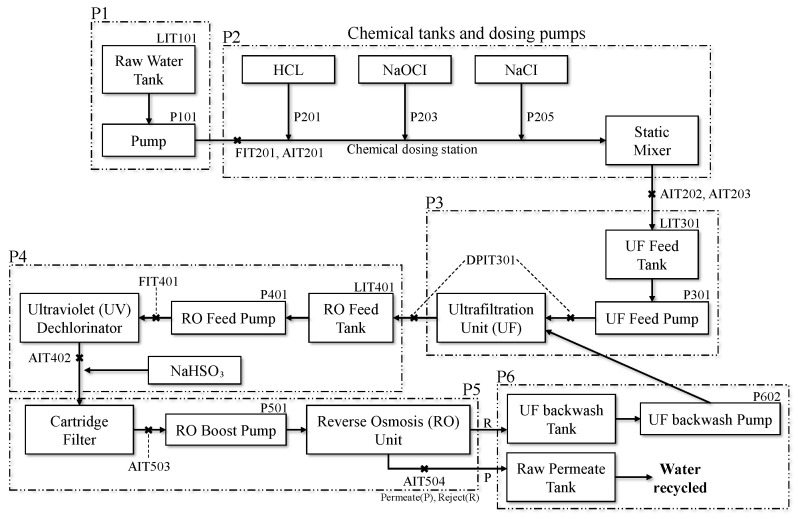
SWaT testbed process flow.

Figure 1 shows the six-stage filtration process of the SWaT testbed, which was designed as a sequential structure. Specifically, the six stages, from P1 to P6, include raw water storage, pretreatment and chemical dosing, ultrafiltration, ultraviolet dechlorination, reverse osmosis, and discharge and recirculation. Each stage is monitored and controlled by industrial sensors, actuators, and PLCs and is centrally managed through the SCADA system and HMI. These 51 sensors and actuators generate the physical process data in the SWaT dataset, which directly reflects the operational state of the plant.

**Figure 2:**
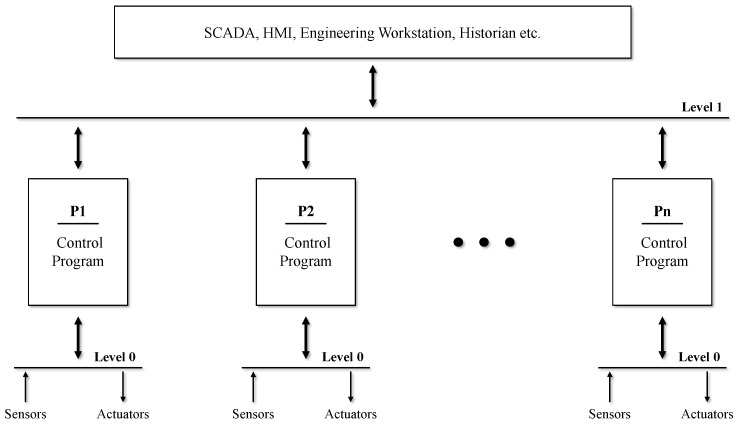
SWaT testbed architecture and control flow.

Figure 2 shows the control and hierarchical communication architecture of the SWaT testbed. At Level 0, sensors and actuators are connected to the PLCs of each process stage, while at Level 1, the SCADA system, HMI, engineering workstation, and historian communicate with the six PLCs. The publicly available network data correspond to the Level 1 communication logs collected from this wired environment and reflect packet exchanges between the SCADA system and the PLCs.

Data collection began after the plant was initialized in an empty state and stabilized under normal operating conditions. Operational data were recorded over approximately 11 days of continuous operation, of which the first seven days corresponded to normal operation data and the following four days included data collected under various cyber and physical attacks. Thirty-six attack scenarios exist, which are categorized into four groups: single-stage single-point (SSSP), single-stage multi-point (SSMP), multi-stage single-point (MSSP), and multi-stage multi-point (MSMP). The attack durations ranged from several minutes to several tens of minutes. These attacks were designed to directly affect process stability and water quality, including sensor spoofing, the forced control of valves and pumps, and chemical dosing disturbances.

### 3.2. Data Quality Assessment

Before constructing the experimental splits, the dataset was assessed in terms of its suitability for sensor and network multimodal anomaly detection. The assessment focused on three aspects: modality coverage, temporal consistency, and label reliability. The dataset provides both physical process measurements and network traffic records, which makes it appropriate for analyzing anomalies from both process-level and communication-level perspectives. In addition, the physical process data are recorded at a regular one-second interval, providing a stable reference timeline for aligning network-level observations.

The attack annotations were also considered reliable for the purpose of this study because the attacks were conducted in a controlled testbed environment and documented with their start and end times, attack points, and intended effects. These records allow anomaly labels to be assigned based on predefined attack intervals rather than inferred after model evaluation. However, the dataset still requires careful temporal handling because the process starts from an initial stabilization period and some attacks may have delayed or persistent physical effects. Therefore, this study uses chronological data splitting and explicit timestamp alignment to reduce temporal inconsistency and prevent information leakage between training, calibration, and test stages.

### 3.3. Dataset Selection and Split

In this study, the SWaT dataset (A1 & A2, 2015) was used for ICS anomaly detection. SWaT is a publicly available dataset collected from a testbed that reflects a real industrial process. As discussed above, its simultaneous availability of physical process data and network logs makes it suitable for the sensor–network multimodal anomaly detection setting considered in this study.

This study followed an unsupervised anomaly detection configuration. Accordingly, data splitting was configured to use only normal data for model training and initial validation and to use data containing attacks for testing and operational threshold adjustment. The same time-based splitting criteria were applied to the sensor and network data throughout the entire preprocessing process. Table 1 lists the period, data composition, purpose, number of attack events, and attack rate for each split.

This splitting design reflects the temporal structure of the SWaT attack logs. According to the publicly available final list of attacks, the first attack began on 28 Dec 2015 at 10:29:14. Therefore, training and validation can be performed using only normal data by separating the period before 28 Dec 2015 at 10:00:00 as normal data. This maintains the fundamental assumptions of unsupervised learning and prevents the attack patterns from contaminating the training process. Furthermore, the operating threshold can be selected without using the test split by separating the threshold calibration and testing periods.

## 4. Proposal of an Unsupervised Anomaly Detection Framework

### 4.1. Overall Framework

**Figure 3:**
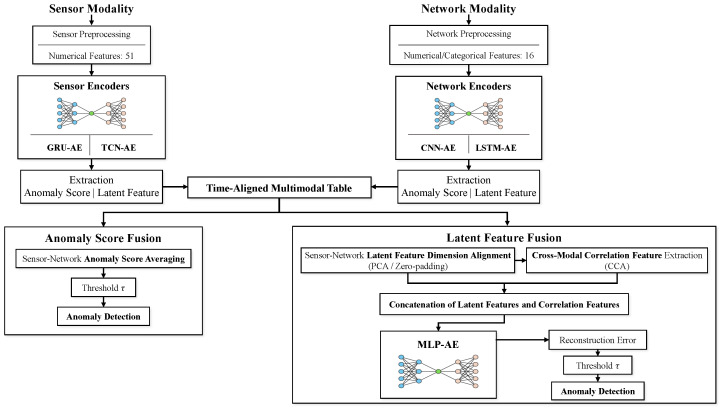
Overview of the unsupervised anomaly detection framework.

In this study, unsupervised learning using an autoencoder (AE) was performed separately for the sensor and network modalities. This study developed a framework that synchronizes and combines the information extracted from the two modalities based on a common time axis and ultimately performs multimodal anomaly detection. Figure 3 illustrates the overall proposed framework.

First, the sensor data and network data from the SWaT dataset were preprocessed separately according to the characteristics of each modality. Next, single-modality unsupervised anomaly detection was performed by applying gated recurrent unit-AE (GRU-AE) and TCN-AE to the sensor modality and convolutional neural network-AE (CNN-AE) and LSTM-AE to the network modality. At this stage, the anomaly scores and latent feature vectors are extracted from each single-modality model.

These outputs are then aligned at a one-second temporal resolution to construct a time-aligned multimodal table, based on which two multimodal detection paths are formed. The first is an anomaly score fusion path in which scores from the sensor and network modalities are combined at the final stage. The second is a latent feature fusion path that aligns the dimensions of the sensor and network latent feature vectors using principal component analysis (PCA) or zero-padding and combines these modality-specific latent features with cross-modal correlation features extracted using canonical correlation analysis (CCA). The aligned latent features and correlation features are then concatenated and fed into an MLP-AE, which produces a reconstruction error for anomaly scoring. In this study, the latent feature fusion-based framework was emphasized as the final proposed framework.

### 4.2. Modality Data Preprocessing

#### 4.2.1. Sensor Data Preprocessing

For the sensor modality, all 51 process variable columns except the timestamp and label were used as features without any additional selection. This was intended to learn representations of physical process information while preserving the relationships among process variables as much as possible. Each feature was converted into a numerical form, and NaN, inf, and -inf values that could occur during preprocessing were corrected by linear interpolation and forward/backward filling.

The data were then divided into four splits based on time: training, validation, calibration, and test. The split criteria followed the date boundaries described in the previous section. For normalization, the minimum and maximum values were calculated using only the training split, and the same Min–Max scaling was then applied to all splits. After normalization, each feature value was clipped to the range [0, 1]. This setting was intended to prevent information leakage from the test data in the unsupervised anomaly detection setting while maintaining a consistent scale across all splits.

The normalized sensor time series were finally reconstructed using a sliding window approach and used as input to the single-modality models. In this study, the window size was set to 12, and the stride was set to 1. Each window was labeled as 1 if it contained at least one attack time point and 0 otherwise. In addition, the representative timestamp for each window was defined as the last time point of the window. This configuration was adopted to preserve local temporal patterns in the sensor time series while reflecting windows containing attacks as anomalous samples.

#### 4.2.2. Network Data Preprocessing

For the network modality, features meaningful for anomaly detection were extracted from SCADA–PLC communication logs by separating them into categorical and numerical features. Seven categorical fields were selected: src, dst, proto, Modbus_Function_Code, s_port, SCADA_Tag, and i/f_dir. The numerical features were defined as a nine-dimensional vector comprising pps, mv_last5, mv_mean, mv_max, and mv_diff. Here, mv_last5 denotes the five most recent values obtained by decoding the hex byte dump of Modbus_Value as little-endian float32, while mv_mean, mv_max, and mv_diff are summary statistics calculated from these values. This feature configuration was designed to reflect simple header information and numerical changes in the Modbus payload.

The categorical features were integer-encoded using a vocabulary constructed from the training split. In particular, for high-cardinality fields, such as src, dst, s_port, and SCADA_Tag, a Top-K vocabulary strategy was adopted to control sparsity and dimensional growth. For the numerical features, the minimum and maximum values were computed from the training split, and the same Min–Max scaling was then applied to all splits. The normalization parameters and vocabularies were stored in JSON files to ensure consistent use in the subsequent training and extraction stages of the single-modality network models.

The normalized network events were finally reconstructed into fixed-length sequences and used as input to the single-modality network models. In this study, the sequence length, step size, and representative timestamp of each sequence were set to 150, 100, and the timestamp of the last event, respectively. A sequence was labeled as 1 if the proportion of attack events within the sequence was at least 5% or if the length of consecutive attack events was at least 3. This criterion was intended to reduce noisy anomaly labeling at the individual-event level and to define anomalous samples as sequences containing a sufficiently meaningful attack segment.

### 4.3. Single-Modality Encoders for Unsupervised Anomaly Detection

In this study, separate single-modality encoders were constructed for the sensor and network modalities because their data characteristics are substantially different. The overall detection principle followed an autoencoder-based unsupervised anomaly detection approach: the model learned normal reconstruction patterns from normal data and then identified samples with large discrepancies between the input and the reconstructed output as anomalies. Accordingly, GRU-AE and TCN-AE were applied to the sensor modality, while CNN-AE and LSTM-AE were applied to the network modality. Each model served as a single-modality anomaly detector and provided latent features for the subsequent multimodal fusion stage.

#### 4.3.1. Sensor Encoders: GRU-AE and TCN-AE

A GRU is a recurrent neural network that learns temporal dependencies in time-series data using update and reset gates within a relatively simple architecture [23]. In this study, the GRU-AE encoded the input sequence using an encoder GRU, projected the final hidden state into a low-dimensional latent representation space, and then used a decoder GRU to reconstruct the full sequence from this representation. The decoder first restored the initial hidden state and context from the latent feature vector and then reconstructed the original time series using a start token and repeated context as inputs. This architecture was well-suited to represent normal patterns compactly while reflecting the temporal context of process sensor data [24].

A TCN is a time-series model that efficiently secures a wide receptive field using dilated convolutions and residual connections [25]. In this study, the TCN-AE encoded the input time series with a TCN encoder, compressed it into a bottleneck representation via a 1 × 1 convolution, and then reconstructed it back to the original input-dimensional time series in the decoder. This was well-suited for learning structural patterns in multivariate sensor time series because it could capture long-term dependencies without using a recurrent structure [26].

#### 4.3.2. Network Encoders: CNN-AE and LSTM-AE

The network modality is a mixed time series containing categorical and numerical fields. Therefore, this study designed sequence-based autoencoders that can process both types of inputs together. Each categorical channel was embedded separately and combined with the normalized numerical features to construct a common input vector.

A CNN is a neural network that extracts local patterns and hierarchical features from input data through convolution operations [27]. In this study, the CNN-AE was designed based on one-dimensional convolutions to model the temporal dependencies along the time axis. The encoder progressively compressed the time-series features through Conv1d blocks, while the decoder reconstructed the original sequence using ConvTranspose1d blocks. The final output consisted of per-channel logits for the categorical channels and reconstructed numerical values. This architecture is advantageous for effectively capturing change patterns across adjacent temporal regions of network events [28].

An LSTM is a model that stably maintains long-term information through input gates, output gates, forget gates, and cell states [29]. In this study, the LSTM-AE was implemented as a seq2seq architecture in which the mixed-input sequence is fed into an encoder LSTM and the full sequence is reconstructed through a decoder LSTM. The decoder input was generated using a teacher-forcing scheme shifted in one time step, and the model finally output the per-channel logits for the categorical channels and reconstructed numerical values. This structure is suitable for reflecting the sequential context and long-term dependencies of network time series.

#### 4.3.3. Anomaly Score and Latent Feature Vector Extraction

In this study, anomaly scores and latent feature vectors for multimodal fusion were extracted from each single-modality encoder. During the extraction stage, the raw anomaly score $s_{\mathrm{raw}}$, raw latent vector $z_{\mathrm{raw}}$, label *y*, and timestamp ts were stored for each split and used later for temporal synchronization and fusion input construction.

For the sensor modality, the GRU-AE uses the vector obtained by projecting the final hidden state of the encoder through a linear layer as its latent feature vector, and the dimensionality of this latent feature vector is 32. The TCN-AE uses the bottleneck representation obtained through a 1 × 1 convolution after the encoder. During extraction, this bottleneck feature map is averaged over the temporal axis to construct a latent feature vector, whose dimensionality is 16. For both models, the anomaly score was defined by calculating the mean squared error (MSE) between the input time series and the reconstructed time series on a per-sample basis.

For the network modality, the CNN-AE uses the global average pooling output to the final feature map of the encoder as its latent feature vector, and its dimensionality is 256. The LSTM-AE uses the final hidden state of the encoder as its latent feature vector, also with a dimensionality of 256. For both models, the anomaly score was defined by combining the reconstruction errors of the categorical channels and the numerical features and then aggregating them over the temporal dimension to obtain a sample-level score.

The anomaly scores and latent feature vectors obtained in this way were subsequently synchronized at a one-second temporal resolution to construct a time-aligned multimodal table, which was then used as the input for anomaly score fusion and latent feature fusion. Therefore, the single-modality encoders in this study served as independent anomaly detectors and feature extractors for multimodal fusion.

### 4.4. Sensor–Network Timestamp Synchronization

The number of samples and their temporal distributions generated at the single-modality stage do not match because the sensor and network modalities undergo different preprocessing and sequence construction procedures. Therefore, an alignment process based on a common temporal reference is required to combine the two modalities. In this study, the timestamp ts recorded during the single-modality extraction stage was converted to a second-level resolution, and samples within the same second were aggregated to construct a time-aligned multimodal table. Multiple samples may correspond to the same second because the network modality uses an event-count-based sequence construction method. Therefore, for the network modality, the raw anomaly score $s_{\mathrm{raw}}$ and raw latent feature vector $z_{\mathrm{raw}}$ were aggregated by their mean values, while label *y* was aggregated by its maximum value, so that the outputs could be aligned with those of the sensor modality at the same timestamp. After this aggregation, only the timestamps shared by both modalities were retained to construct the final multimodal table.

The final time-aligned multimodal table stores the sensor anomaly score $s^{\mathrm{sen}}$, the network anomaly score $s^{\mathrm{net}}$, the sensor latent feature vector $z^{\mathrm{sen}}$, the network latent feature vector $z^{\mathrm{net}}$, and the final label *y* and the timestamp sec. Through this structure, the sensor and network modalities can be compared and combined on the same time axis, and the resulting table is used as the common input for anomaly score fusion and latent feature fusion.

Table 2 lists the number of samples in each split before and after temporal synchronization. Here, the number of sensors and network samples refers to the raw row-level samples obtained from each single-modality extraction result. The number of multimodal samples refers to the result after second-level aggregation, retaining only the timestamps shared by both modalities. Because the training and validation splits are normal-only segments, the attack ratio of the common samples is 0.00% for both. In contrast, the calibration and test splits show attack ratios of 4.53% and 13.13%, respectively.

### 4.5. Multimodal Anomaly Detection via Anomaly Score Fusion

This section describes the multimodal anomaly detection framework based on anomaly score fusion. This is a late-fusion approach in which scores produced from each modality are combined to generate a final score. Hence, it uses the outputs of the single-modality detectors without any additional feature extraction or fusion of latent vectors. The input to anomaly score fusion consists of the sensor and network scores in the time-aligned multimodal table after temporal alignment. These scores correspond one-to-one on the same time axis. Let $s_{t}^{\mathrm{sen}}$ denote the sensor score and $s_{t}^{\mathrm{net}}$ denote the network score at time *t*. The final fused score $s_{t}^{\mathrm{ASF}}$ is then defined by Equation (1):

(1) $$s_{t}^{\mathrm{ASF}}=\frac{s_{t}^{\mathrm{sen}}+s_{t}^{\mathrm{net}}}{2}.$$

In this study, anomaly score fusion uses a simple arithmetic mean that equally weights the scores from the two modalities. That is, no separate weighting parameter is introduced, and the signals provided by the two modalities are treated as equally important. This setup was employed to ensure a parameter-free and reproducible score fusion, thereby bypassing manual adjustments and calibration-dependent weights. While adaptive weights could be an alternative, this approach requires additional criteria for determining the weights. Specifically, learning weights based on detection performance relies on anomaly labels. This contradicts the premise of an unsupervised environment, rendering the fusion path label-dependent. Furthermore, to achieve fully unsupervised adaptive weighting, a separate mechanism is needed to estimate the confidence of each modality. Therefore, this work adopts equal weighting to impartially assess the effect of directly combining sensor and network anomaly scores. The fused score is then converted into a binary decision based on a threshold τ. The final decision function is given by Equation (2):

(2) $${\hat{y}}_{t}=\left\{\begin{array}{cc} 1, & s_{t}^{\mathrm{ASF}}\geq \tau \\ 0, & s_{t}^{\mathrm{ASF}}<\tau \end{array}\right..$$

Here, ${\hat{y}}_{t}$ is the final predicted label at time *t*, where 1 indicates an anomaly, and 0 indicates normality. The generated fusion scores are stored for each split and later used to calculate the evaluation metrics, such as F1-score, FPR, and AUC, based on the operating threshold determined through the threshold selection procedure described in Section 5.2.

Within the overall framework, anomaly score fusion provides a simple late fusion path for combining modality-specific detection results. This approach is straightforward because it does not require additional model training. However, because it combines only the final anomaly score information, it cannot explicitly capture cross-modal information between the sensor and network modalities.

### 4.6. Multimodal Anomaly Detection via Latent Feature Fusion

The core contribution of this study is a multimodal detection framework based on latent feature fusion. Unlike late fusion, which combines the final anomaly scores from each modality, this approach uses the internal latent feature vectors extracted from the sensor and network encoders. This design enables the framework to reflect both modality-specific latent information and cross-modal relationships before reconstruction-based anomaly detection is performed.

Let $z_{t}^{\mathrm{sen}}\in R^{d_{s}}$ denote the sensor latent feature vector and $z_{t}^{\mathrm{net}}\in R^{d_{n}}$ denote the network latent feature vector at time *t*. Because sensor and network encoders can produce latent vectors with different dimensions, the two latent features are first transformed to the same target dimension *d* through modality-specific standardization and dimensional alignment. In this study, d=32. When the input dimension equals the target dimension, the latent vector is kept unchanged; if it is larger, PCA-based dimensionality reduction is applied, whereas if it is smaller, zero-padding is used to match the target dimension.

PCA and zero-padding were adopted as lightweight dimensional alignment methods to reduce unnecessary distortion of modality-specific latent information. The purpose of this step is not to learn a new fusion feature by itself but to place the sensor and network latent vectors in a comparable dimensional form while preserving the information extracted by each single-modality encoder as much as possible. Consequently, the aligned latent vectors of the sensor and network are denoted as ${z_{t}^{\mathrm{sen}}}_{\mathrm{aligned}}\in R^{d}$ and ${z_{t}^{\mathrm{net}}}_{\mathrm{aligned}}\in R^{d}$, respectively.

After dimensional alignment, cross-modal correlation features are extracted using CCA. CCA is a method that finds a pair of linear projections for two related data views such that the projected components are maximally correlated. In this study, CCA is used to identify latent directions in which the sensor and network modalities exhibit related behavior under the common time axis. Let $c_{t}^{\mathrm{sen}}\in R^{d_{cca}}$ and $c_{t}^{\mathrm{net}}\in R^{d_{cca}}$ denote the CCA-derived correlation features obtained from the aligned sensor and network latent vectors, respectively. In this study, $d_{cca}=16$.

The CCA-derived features provide cross-modal correlation information, but they are not used alone. Because CCA mainly captures shared linear correlation structures, relying only on CCA-derived features may attenuate modality-specific anomaly patterns that appear strongly in either the sensor or network modality. Therefore, the dimension-aligned sensor and network latent vectors are retained together with the CCA-derived correlation features. This design allows the fused feature to include both modality-specific latent evidence and cross-modal correlation information.

The final latent feature fusion is constructed by concatenating four latent components: the aligned sensor latent feature, the aligned network latent feature, the CCA-derived sensor-side correlation feature, and the CCA-derived network-side correlation feature. This is expressed as Equation (3):

(3) $$z_{t}^{\mathrm{LFF}}=\left[{z_{t}^{\mathrm{sen}}}_{\mathrm{aligned}}\;;\;{z_{t}^{\mathrm{net}}}_{\mathrm{aligned}}\;;\;c_{t}^{\mathrm{sen}}\;;\;c_{t}^{\mathrm{net}}\right].$$

The fused feature has dimensionality $2d+2d_{cca}$. In this study, d=32 and $d_{cca}=16$, resulting in a 96-dimensional fused latent feature. The constructed fused feature vector $z_{t}^{\mathrm{LFF}}$ is used as the input to an MLP-based autoencoder. The autoencoder encodes the fused feature into a lower-dimensional bottleneck and then decodes it to reconstruct the original fused feature. In this study, a structure was used that compresses the 96-dimensional input into a 16-dimensional bottleneck.

The final anomaly score is defined as the MSE between the fused latent feature and its reconstruction. A smaller reconstruction error indicates that the fused sensor–network latent pattern at that time step is close to the learned normal pattern, whereas a larger reconstruction error suggests that the modality-specific latent information or the cross-modal correlation structure deviates from the learned normal behavior [30]. Training is performed using only normal samples, and the final anomaly decision is made based on the threshold τ, as expressed in Equation (4).

(4) $${\hat{y}}_{t}=\left\{\begin{array}{cc} 1, & s_{t}^{\mathrm{LFF}}\geq \tau \\ 0, & s_{t}^{\mathrm{LFF}}<\tau \end{array}\right..$$

Therefore, latent feature fusion enables multimodal anomaly detection by modeling a fused latent feature that preserves modality-specific latent features while incorporating CCA-derived cross-modal correlation features. This allows the MLP-AE to learn normal sensor–network patterns from both individual modality evidence and their correlated behavior under the aligned time axis.

## 5. Experiments of Proposed Anomaly Detection

### 5.1. Experimental Environment

The SWaT dataset (A1 & A2, 2015), which was collected at the iTrust Centre of the Singapore University of Technology and Design (SUTD), was used to evaluate the proposed framework. In the single-modality experiments, GRU-AE and TCN-AE were used for the sensor modality, while CNN-AE and LSTM-AE were used for the network modality. Four combinations were constructed in the multimodal experiments: GRU-CNN, GRU-LSTM, TCN-CNN, and TCN-LSTM. For each combination, anomaly score fusion and latent feature fusion were evaluated separately.

The multimodal experiments were conducted after aligning the anomaly scores and latent feature vectors of the two modalities on a one-second time axis and constructing common time points because the values extracted from the single-modality sensor and network models have different temporal distributions. After temporal synchronization, the number of common samples was 371,238 for training, 122,389 for validation, 125,875 for calibration, and 289,288 for testing.

In latent feature fusion, additional dimensional alignment was applied because the dimensions of the latent feature vectors differed across modalities. The target dimension for the modality-specific aligned latent features was set to 32. For the sensor modality, the 32-dimensional features from GRU-AE were used as is, while the 16-dimensional features from TCN-AE were expanded to 32 dimensions via zero-padding. For the network modality, PCA was applied to the 256-dimensional features from the CNN-AE and LSTM-AE to reduce them to 32 dimensions. After dimensional alignment, 16-dimensional CCA-derived correlation features were additionally extracted from each modality. Therefore, the final latent feature fusion input consisted of four components: the aligned sensor latent feature, the aligned network latent feature, the sensor-side CCA-derived correlation feature, and the network-side CCA-derived correlation feature. As a result, the final fusion input was constructed as a 96-dimensional vector.

Table 3 lists the main settings of each single-modality model and the latent feature fusion model. For reference, anomaly score fusion was excluded from the table because it did not involve additional model training.

### 5.2. Evaluation Metrics and Threshold Selection

In this study, the Precision, Recall, F1-score, Precision–Recall Area Under the Curve (PR-AUC), Receiver Operating Characteristic Area Under the Curve (ROC-AUC), and False Positive Rate (FPR) were used as the evaluation metrics for anomaly detection performance. Precision, Recall, F1-score, and FPR were used to assess the detection characteristics under a selected operating threshold, whereas PR-AUC and ROC-AUC were used to evaluate the overall separability of the models. In particular, in ICS environments, false alarms during normal operation directly affect the system reliability. Therefore, high separability and the ability to achieve stable detection under low-false-positive conditions must be considered.

The main evaluation in this study was conducted based on threshold selection using the validation and calibration data. First, a minimum threshold was determined from the normal validation split to ensure that the false-positive rate did not exceed 1%. Next, an additional threshold floor was calculated using the normal samples in the calibration split under a stricter false-positive constraint. This step was used to prevent the selected threshold from producing excessive false alarms on normal calibration samples. Among the remaining candidates, the final operating threshold was selected by prioritizing higher recall, followed in order by higher precision, lower calibration-normal FPR, lower overall calibration FPR, and a lower threshold. This calibration-based selection was used to determine a practical operating point that considers both false-alarm control and attack detection sensitivity, rather than relying only on normal-score distributions.

The calibration split was used only for operating-threshold selection and was temporally separated from the final test split. The test split was not used for threshold selection, model tuning, or any calibration step. After the operating threshold was fixed, it was applied once to the test split for final evaluation. Therefore, the reported Precision, Recall, F1-score, and FPR correspond to the final test performance under the preselected operating threshold, while the ROC-AUC and PR-AUC were computed from the continuous anomaly scores on the test split.

### 5.3. Detection Performance

Table 4 lists the results of single-modality anomaly detection. In the sensor modality, GRU-AE outperformed TCN-AE, whereas CNN-AE outperformed LSTM-AE in the network modality. Overall, the F1-scores were somewhat limited, but the separability between normal and anomalous samples was relatively strong, as measured by the ROC-AUC and PR-AUC. This suggests that, although single-modality approaches can achieve a meaningful level of anomaly-score separability, there is still room to improve the final binary detection performance through multimodal fusion.

Table 5 lists the multimodal detection results of anomaly score fusion. Anomaly score fusion combines the final anomaly scores of the two single-modality detectors and provides a late-fusion path within the proposed framework. The results show that anomaly score fusion can improve detection performance in some model combinations, but its effectiveness varies depending on the score distributions of the individual sensor and network detectors.

Table 6 lists the results of latent feature fusion. For this experiment, the proposed fusion path was repeated using five fixed random seeds to examine its stability. The five seeds were sampled once using a fixed seed-selection procedure and then applied consistently to all model combinations. The results are reported as the mean and standard deviation across the repeated runs.

Overall, the latent feature fusion results showed consistently high detection performance across the four sensor–network encoder combinations. Among them, the GRU-AE and CNN-AE combination achieved the best overall performance in terms of the F1-score, ROC-AUC, and PR-AUC. The TCN-AE and LSTM-AE combination achieved the lowest FPR while maintaining comparable F1-score performance. In contrast, the TCN-AE and CNN-AE combination showed larger variation across seeds, especially in Recall and F1-score. These results indicate that the performance of latent feature fusion can vary depending on the selected sensor–network encoder pair.

Table 7 compares the proposed LFF with related multimodal ICS anomaly detection studies. The compared studies include different types of multimodal fusion strategies, such as early feature fusion, late decision fusion, cross-domain fusion, and cross-attention fusion. Because the reported metrics differ across studies, the comparison focuses on commonly available threshold-dependent metrics, including Precision, Recall, F1-Score, and FPR. Because the datasets, preprocessing strategies, labeling criteria, and evaluation protocols are not identical, the results should be interpreted as a contextual numerical comparison rather than a strictly controlled benchmark. In this comparison, the proposed LFF achieved the highest Precision and F1-Score, as well as the lowest FPR, while maintaining competitive Recall.

The methodological strength of the proposed LFF lies in its lightweight and modular fusion strategy, differentiating it from intermediate fusion approaches such as cross-domain and cross-attention fusion methods. Cross-domain fusion explicitly models cross-modal relationships through graph structures, and cross-attention fusion learns flexible cross-modal interactions. Therefore, both fusion methods are highly expressive. However, this expressiveness can introduce additional computational complexity. Furthermore, these approaches tend to focus heavily on complex cross-modal interactions, which may occasionally attenuate the anomaly evidence inherent in each individual modality. In contrast, the proposed LFF preserves modality-specific latent features extracted via independently trained single-modality encoders and incorporates CCA-derived correlation features to capture shared linear relationships. This design enables the model to leverage both individual modality evidence and cross-modal correlation information without relying on complex interaction-learning modules.

### 5.4. Result Analysis and Practical Considerations

#### 5.4.1. Cross-Modal Complementarity

**Figure 4:**
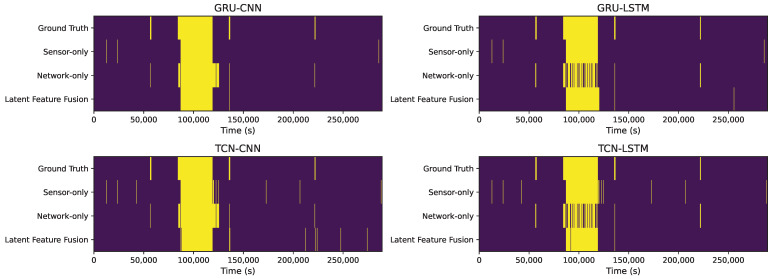
Anomaly detection results for four fusion pairs, showing ground truth, single-modality models, and latent feature fusion along the aligned-time axis.

Figure 4 shows how cross-modal complementarity emerges between the sensor and network modalities via latent feature fusion. This figure visualizes binary detection results on the aligned one-second time axis. Here, ’Ground Truth’ indicates the true attack intervals, ’Sensor-only’ and ’Network-only’ denote the anomaly decisions of the corresponding single-modality models, and ’Latent Feature Fusion’ denotes the anomaly decisions of the proposed fusion model. Yellow indicates attack or detected anomaly points, whereas purple indicates normal points. The sensor and network models did not produce identical detection patterns over the attack intervals, and their response timing also differed within the same interval. This indicates that the two modalities respond to anomalous behavior in different ways.

Table 8 further quantifies this complementarity by dividing attack samples into Both hit, Sensor only, Network only, and Both miss groups. The Network only group appeared at a similar level across all pairs, whereas the Sensor only group varied substantially depending on the network encoder, indicating that the degree and direction of complementarity depend on the sensor–network encoder combination. The recovery results show that LFF detected a portion of the missed attack samples, but the recovered modality differed by encoder pair: GRU-LSTM and TCN-LSTM showed high recovery rates for network misses, while TCN-CNN showed the highest recovery rate for sensor misses and both misses. This suggests that the contribution of LFF changes according to the latent representations used in fusion.

Overall, this complementarity analysis supports the motivation for latent feature fusion. The proposed LFF does not simply reproduce the decisions of the single-modality detectors. Instead, it constructs a fused latent representation that combines modality-specific latent features with cross-modal correlation information. Therefore, LFF is meaningful because sensor and network modalities provide partially different anomaly evidence, and their integration can improve the robustness of multimodal anomaly detection.

#### 5.4.2. Real-Time Deployment and Computational Complexity

In practical ICS environments, anomaly detection models should introduce limited computational overhead in addition to achieving high detection performance. In the proposed framework, several computationally expensive procedures are performed offline. In particular, PCA and CCA fitting are conducted using the training split and are not repeated during online detection.

During online detection, the LFF stage applies the stored standardization, PCA, and CCA projection parameters; constructs the 96-dimensional fused latent representation; computes the reconstruction error using the trained fusion MLP-AE; and compares the resulting anomaly score with the selected threshold. The fusion MLP-AE used in this study has a compact structure with a 96-dimensional input, 64 hidden units, a 16-dimensional bottleneck, and 14,576 trainable parameters.

The measured fusion-stage runtime also indicates a small online overhead. Across the four encoder combinations, the average amortized processing time was approximately 0.000064–0.000071 ms per sample on the test split under batched GPU inference. Therefore, the additional cost introduced by the LFF stage is small in the experimental setting used in this study. However, this measurement should be interpreted as the cost of the fusion stage only, rather than the complete end-to-end deployment cost. In real ICS environments, additional overhead may arise from packet parsing, sensor data acquisition, feature extraction, timestamp alignment, and monitoring-system integration.

#### 5.4.3. Discussion of Limitations

Although the proposed framework showed effective multimodal anomaly detection performance, several limitations should be acknowledged. First, the framework depends on temporal alignment between sensor and network data. In this study, both modalities were aligned on a common one-second time axis, which enabled sample-level multimodal fusion. However, in practical ICS environments, timestamp errors, communication delays, packet losses, or different sampling rates may degrade the quality of temporal alignment and affect fusion performance.

Second, the proposed framework follows a normal-only training assumption, which was satisfied in the experimental design by constructing the training split from normal operation periods only. However, in real deployments, training data may contain unnoticed abnormal samples, and normal behavior itself may change over time due to process drift, maintenance, or configuration changes. Therefore, future work should consider contamination-robust training or adaptive updating strategies.

Third, the dimensional alignment strategy was designed to be lightweight and modular by combining PCA, zero-padding, and CCA-derived correlation features. Although this design preserves modality-specific latent features and supports effective fusion in this study, its robustness should be further validated under different encoder architectures, latent dimensions, and ICS datasets.

Fourth, anomaly detection performance was evaluated in the overall attack intervals rather than separately for the four SWaT attack categories: SSSP, SSMP, MSSP, and MSMP. Although these results validate the effectiveness of the proposed multimodal fusion approach, an attack-type-level analysis could clarify which categories benefit most from sensor–network fusion. This suggests that further work should analyze how multimodal fusion contributes to detection performance for each attack category.

Finally, detecting attacks that leave weak traces in both modalities remains challenging. As shown in Table 8, LFF recovered a portion of the attacks missed by individual single-modality models, but the recovery of attacks missed by both modalities was limited in some encoder combinations. This suggests that, when both sensor and network representations provide weak anomaly evidence, additional temporal context, event-level aggregation, or more expressive fusion mechanisms may be needed.

## 6. Conclusions

In ICS environments, anomalies may manifest differently across physical process data and network communication data, which makes single-modality detection inherently limited. This study developed an unsupervised multimodal anomaly detection framework for ICS environments that jointly utilizes sensor and network modalities, and systematically compared two time-synchronization-based fusion strategies: anomaly score fusion and latent feature fusion.

The experimental results showed that anomaly score fusion improved detection performance in some model combinations, but its effectiveness varied depending on the score distributions of the individual sensor and network detectors. In contrast, latent feature fusion provided more stable multimodal detection performance by integrating modality-specific latent features and cross-modal correlation information, with the GRU-AE and CNN-AE combination achieving the best overall results. In addition, the time-aligned detection results and miss-recovery analysis showed that sensor and network modalities responded differently to attack samples and that latent feature fusion could recover a portion of the attacks missed by individual modalities. These findings indicate that multimodal fusion performance is determined not only by the individual strength of each modality but also by how effectively complementary information is integrated.

Overall, the results demonstrate that latent feature fusion is a meaningful and practical fusion path for multimodal ICS anomaly detection. Future work should further validate the proposed framework on additional ICS datasets and conduct attack-type-level detection performance analysis. In addition, more adaptive fusion strategies should be investigated for environments with temporal misalignment, evolving normal behavior, or weak anomaly traces in both modalities. Such extensions are expected to provide a more comprehensive understanding of multimodal fusion and support more robust deployment in practical ICS environments.

## Figures and Tables

**Table 1:** Data split configuration and attack ratio.

Split	Period	Data Composition	Purpose	Attack Events	Attack Ratio (%)
Train	22 Dec 2015–26 Dec 2015	Normal-only	Model training	0	0.00
Validation	27 Dec 2015–28 Dec 2015 09:59:59	Normal-only	Initial validation	0	0.00
Calibration	28 Dec 2015 10:00:00–29 Dec 2015	Normal + Attack	Operating threshold calibration	17	5.82
Test	30 Dec 2015–2 Jan 2016	Normal + Attack	Performance evaluation	19	13.29

**Table 2:** Number of multimodal samples for each split after temporal alignment.

Split	Sensor Samples	Network Samples	Multimodal Samples	Attack Ratio (%)
Training	372,589	792,637	371,238	0.00
Validation	122,389	224,367	122,389	0.00
Calibration	136,789	230,735	125,875	4.53
Test	313,108	592,973	289,288	13.13

**Table 3:** Model configuration.

Model	Configuration
Sensor GRU-AE	$h_{s}$ = 64, *z* = 32, *L* = 1, *B* = 256, *p* = 0.0
Sensor TCN-AE	$h_{c}$ = 64, *b* = 16, lev = 3, *k* = 3, *B* = 256, *p* = 0.1
Network CNN-AE	$h_{c}$ = 256, *L* = 3, *k* = 5, *B* = 128, *p* = 0.1
Network LSTM-AE	$h_{s}$ = 256, *L* = 1, *B* = 128, *p* = 0.0
Latent Feature Fusion MLP-AE	in = 96, $h_{d}$ = 64, *b* = 16, *B* = 512, *p* = 0.1

$h_{s}$, $h_{c}$, and $h_{d}$ denote hidden size, hidden channels, and hidden dimension, respectively; *z* denotes latent dimension; *b* bottleneck dimension; in input dimension; *L* the number of layers; lev the number of temporal levels; *k* kernel size; *B* batch size; and *p* dropout rate.

**Table 4:** Performance comparison of single-modality anomaly detection models.

Modality	Model	F1-Score	FPR	PR-AUC	ROC-AUC
Sensor	GRU-AE	0.7014	0.0755	0.8363	0.9100
Sensor	TCN-AE	0.5515	0.1795	0.7531	0.8878
Network	CNN-AE	0.8521	0.0402	0.9478	0.9915
Network	LSTM-AE	0.7331	0.0335	0.8001	0.9152

**Table 5:** Performance comparison of multimodal anomaly score fusion models.

Pair	Precision	Recall	F1-Score	FPR	PR-AUC	ROC-AUC
GRU-CNN	0.4803	0.9718	0.6429	0.1589	0.9761	0.9926
GRU-LSTM	0.5995	0.9104	0.7229	0.0919	0.9442	0.9809
TCN-CNN	0.8346	0.9846	0.9034	0.0295	0.9683	0.9941
TCN-LSTM	0.5646	0.9311	0.7029	0.1085	0.9447	0.9826

**Table 6:** Performance comparison of multimodal latent feature fusion models.

Pair	Precision	Recall	F1-Score	FPR	PR-AUC	ROC-AUC
GRU-CNN	0.9729 ± 0.0139	0.8665 ± 0.0066	0.9166 ± 0.0033	0.0037 ± 0.0019	0.9516 ± 0.0037	0.9795 ± 0.0027
GRU-LSTM	0.9528 ± 0.0127	0.8580 ± 0.0047	0.9029 ± 0.0036	0.0065 ± 0.0019	0.9360 ± 0.0066	0.9724 ± 0.0043
TCN-CNN	0.9695 ± 0.0232	0.8405 ± 0.0742	0.8985 ± 0.0421	0.0042 ± 0.0034	0.9365 ± 0.0062	0.9692 ± 0.0034
TCN-LSTM	0.9862 ± 0.0066	0.8509 ± 0.0029	0.9136 ± 0.0040	0.0018 ± 0.0009	0.9241 ± 0.0033	0.9586 ± 0.0055

Values are reported as mean ± standard deviation over five fixed random seeds.

**Table 7:** Performance comparison with related multimodal ICS anomaly detection studies.

Study	Dataset	Fusion Type	Precision	Recall	F1-Score	FPR
Du et al. [18]	WDT	Early feature fusion	0.8000	0.7200	0.7580	–
Canonico et al. [19]	SWaT	Late decision fusion	0.8837	0.9639	0.9162	–
Zhan et al. [20]	SWaT	Cross-domain fusion	0.8465	0.8512	0.8488	0.0307
Pinto et al. [21]	SWaT	Cross-attention fusion	0.8488	0.7898	0.8183	0.0180
Proposed LFF (GRU-AE–CNN-AE)	SWaT	Latent feature fusion	0.9729	0.8665	0.9166	0.0037

Values were unified to the 0–1 scale when necessary. Pinto et al. [21]’s Precision, Recall, and FPR were derived from the reported confusion counts.

**Table 8:** Complementarity analysis of single-modality detections and LFF recovery.

Pair	Both Hit	Sensor Only	Network Only	Both Miss	LFF Recovery of Sensor Misses	LFF Recovery of Network Misses	LFF Recovery of Both Misses
GRU-CNN	31,933 (84.07%)	182 (0.48%)	4104 (10.80%)	1767 (4.65%)	515/5871 (8.77%)	222/1949 (11.39%)	67/1767 (3.79%)
GRU-LSTM	21,233 (55.90%)	10,882 (28.65%)	3865 (10.17%)	2006 (5.28%)	641/5871 (10.92%)	10,888/12,888 (84.48%)	27/2006 (1.35%)
TCN-CNN	32,059 (84.40%)	195 (0.51%)	3978 (10.47%)	1754 (4.62%)	1419/5732 (24.76%)	460/1949 (23.60%)	302/1754 (17.22%)
TCN-LSTM	21,337 (56.17%)	10,917 (28.74%)	3761 (9.90%)	1971 (5.19%)	621/5732 (10.83%)	10,741/12,888 (83.34%)	8/1971 (0.41%)

Both hit, Sensor only, Network only, and Both miss are based on single modality decisions for attack samples. LFF recovery indicates the proportion of attacks missed by each single modality but detected by LFF. LFF recovery of both misses denotes the proportion of attacks missed by both single modality models but detected by LFF.

## Data Availability

Data are contained within the article.

## Abbreviations

The following abbreviations are used in this manuscript:

ICS	industrial control system
PLC	programmable logic controller
DCS	distributed control system
SCADA	supervisory control and data acquisition
HMI	human–machine interface
IIoT	Industrial Internet of Things
LSTM	long short-term memory
TCN	temporal convolutional network
SWaT	secure water treatment
SSAP	single-stage single-point
SSMP	single-stage multi-point
MSSP	multi-stage single-point
MSMP	multi-stage multi-point
AE	autoencoder
GRU	gated recurrent unit
CNN	convolutional neural network
PCA	principal component analysis
CCA	canonical correlation analysis
MSE	mean squared error
PR-AUC	Precision–Recall Area Under the Curve
ROC-AUC	Receiver Operating Characteristic Area Under the Curve
FPR	false positive rate

## References

[1] Bhamare D., Zolanvari M., Erbad A., Jain R., Khan K., Meskin N. (2020). Cybersecurity for industrial control systems: A survey. Comput. Secur..

[2] Mubarak S., Habaebi M.H., Islam M.R., Rahman F.D.A., Tahir M. (2021). Anomaly detection in ICS datasets with machine learning algorithms. Comput. Syst. Sci. Eng..

[3] Abualghanam O., Alazzam H., Almobaideen W. (2025). Hierarchical lightweight intrusion detection system using deep learning in the context of IoT. Clust. Comput..

[4] Koay A.M., Ko R.K.L., Hettema H., Radke K. (2023). Machine learning in industrial control system (ICS) security: Current landscape, opportunities and challenges. J. Intell. Inf. Syst..

[5] Hu Y., Yang A., Li H., Sun Y., Sun L. (2018). A survey of intrusion detection on industrial control systems. Int. J. Distrib. Sens. Netw..

[6] MR G.R., Ahmed C.M., Mathur A. (2021). Machine learning for intrusion detection in industrial control systems: Challenges and lessons from experimental evaluation. Cybersecurity.

[7] Kim H., Shon T. (2022). Industrial network-based behavioral anomaly detection in AI-enabled smart manufacturing. J. Supercomput..

[8] Sinha A., Taylor M., Srirama N., Manikas T., Larson E.C., Thornton M.A. Industrial control system anomaly detection using convolutional neural network consensus. Proceedings of the 2021 IEEE Conference on Control Technology and Applications (CCTA).

[9] Macas M., Wu C. An unsupervised framework for anomaly detection in a water treatment system. Proceedings of the 2019 18th IEEE International Conference on Machine Learning and Applications (ICMLA).

[10] Umer M.A., Junejo K.N., Jilani M.T., Mathur A.P. (2022). Machine learning for intrusion detection in industrial control systems: Applications, challenges, and recommendations. Int. J. Crit. Infrastruct. Prot..

[11] Malhotra P., Vig L., Shroff G., Agarwal P. (2015). Long short term memory networks for anomaly detection in time series. Proceedings.

[12] Gopali S., Abri F., Siami-Namini S., Namin A.S. A comparison of TCN and LSTM models in detecting anomalies in time series data. Proceedings of the 2021 IEEE International Conference on Big Data (Big Data).

[13] Ortega-Fernandez I., Sestelo M., Burguillo J.C., Piñón-Blanco C. (2024). Network intrusion detection system for DDoS attacks in ICS using deep autoencoders. Wirel. Netw..

[14] Zhanwei S., Zenghui L. (2019). Abnormal detection method of industrial control system based on behavior model. Comput. Secur..

[15] Zare F., Mahmoudi-Nasr P., Yousefpour R. (2024). A real-time network based anomaly detection in industrial control systems. Int. J. Crit. Infrastruct. Prot..

[16] Berge V., Li C. (2024). Enhanced Anomaly Detection in Industrial Control Systems aided by Machine Learning. arXiv.

[17] Zhao F., Zhang C., Geng B. (2024). Deep multimodal data fusion. ACM Comput. Surv..

[18] Du Y., Huang Y., Wan G., He P. (2022). Deep learning-based cyber–physical feature fusion for anomaly detection in industrial control systems. Mathematics.

[19] Canonico R., Esposito G., Navarro A., Romano S.P., Sperlì G., Vignali A. (2025). Empowered Cyber–Physical Systems security using both network and physical data. Comput. Secur..

[20] Zhan D., Zhang W., Ye L., Yu X., Zhang H., He Z. (2024). Anomaly detection in industrial control systems based on cross-domain representation learning. IEEE Trans. Dependable Secur. Comput..

[21] Pinto A., Herrera L.C., Donoso Y., Gutierrez J.A. (2026). Cyber-physical anomaly detection a deep adversarial fusion of sensor and network data. Discov. Comput..

[22] Goh J., Adepu S., Junejo K.N., Mathur A. (2016). A dataset to support research in the design of secure water treatment systems. Proceedings of the International Conference on Critical Information Infrastructures Security.

[23] Cho K., Van Merriënboer B., Gulçehre Ç., Bahdanau D., Bougares F., Schwenk H., Bengio Y. Learning phrase representations using RNN encoder–decoder for statistical machine translation. Proceedings of the 2014 Conference on Empirical Methods in Natural Language Processing (EMNLP).

[24] Sutskever I., Vinyals O., Le Q.V. (2014). Sequence to sequence learning with neural networks. Adv. Neural Inf. Process. Syst..

[25] Bai S., Kolter J.Z., Koltun V. (2018). An empirical evaluation of generic convolutional and recurrent networks for sequence modeling. arXiv.

[26] Thill M., Konen W., Wang H., Bäck T. (2021). Temporal convolutional autoencoder for unsupervised anomaly detection in time series. Appl. Soft Comput..

[27] Masci J., Meier U., Cireşan D., Schmidhuber J. (2011). Stacked convolutional auto-encoders for hierarchical feature extraction. Proceedings of the International Conference on Artificial Neural Networks.

[28] Russo S., Disch A., Blumensaat F., Villez K. (2020). Anomaly detection using deep autoencoders for in-situ wastewater systems monitoring data. arXiv.

[29] Hochreiter S., Schmidhuber J. (1997). Long short-term memory. Neural Comput..

[30] Fährmann D., Damer N., Kirchbuchner F., Kuijper A. (2022). Lightweight long short-term memory variational auto-encoder for multivariate time series anomaly detection in industrial control systems. Sensors.

